# Design and Characterization of Epoxy Resin Systems Based on Mixtures of Imidazolium-Based Ionic Liquids with Docusate and Dicyanamide Anions

**DOI:** 10.3390/molecules29194538

**Published:** 2024-09-24

**Authors:** Andrea Szpecht, Dawid Zielinski, Szymon Roszyk, Marcin Smiglak

**Affiliations:** 1 Poznan Science and Technology Park, Adam Mickiewicz University Foundation, 61-612 Poznan, Poland; andrea.szpecht@ppnt.poznan.pl (A.S.); dawid.zielinsk@ppnt.poznan.pl (D.Z.); szymon.roszyk@student.put.poznan.pl (S.R.); 2 Faculty of Chemical Technology, Poznan University of Technology, 60-965 Poznan, Poland

**Keywords:** ionic liquids, epoxy resin, crosslinking

## Abstract

This study focuses on the synthesis, characterization, and application of four ionic liquids (ILs), three of which are being reported for the first time, with unique thermal properties and diverse anion-cation combinations, specifically in the context of epoxy resin polymerization. 1-3-Didodecylimidazolium dicyanamide (dDDIM DCA), 1-3-Didodecylimidazolium docusate (dDDIM DOSS), 1-ethyl-3-methylimidazolium dicyanamide (EMIM DCA), and 1-ethyl-3-methylimidazolium docusate (EMIM DOSS) were used to prepare six different mixtures with the same cation and with varying concentrations of DCA components, which is the main factor of an efficient polymerization, while the other component is intended to modify the properties of the cured resin. Mixtures based on EMIM cation demonstrated increased enthalpy and lower onset polymerization temperatures, indicating more efficient curing processes. The hardness of cured epoxy resins can be adjusted by altering the curing temperature and IL composition, with EMIM DCA and EMIM DOSS mixtures displaying high Shore A hardness, suitable for durable surface applications. In contrast, mixtures with higher dDDIM DCA proportions offered a balance between rigidity and flexibility, ideal for applications requiring both mechanical strength and elasticity.

## 1. Introduction

Ionic liquids (ILs) are defined as salts that consist entirely of ions with a melting point below 100 °C [1]. ILs possess many unique physicochemical properties, combining the advantages of traditional molten salts yet omitting many of their disadvantages. The ionic liquids are characterized by extremely low vapor pressure, temperature-dependent fluidity, high thermal and good chemical stability, low toxicity, high ionic conductivity, and excellent solvent properties for organic and inorganic compounds [2].

The most well-known class of ILs are imidazolium-based ionic liquids, which represent a rapidly growing class of chemicals that capture the interest of researchers from various fields. They are particularly valued for their role as green solvents in chemical reactions and processes, given their non-volatile nature and the ability to be recycled [3]. Additionally, the tunability of both the imidazolium and anionic components allows for the design of ionic liquids with specific properties tailored to applications, such as catalysis [4], electrochemistry [5], and materials synthesis [6]. Beyond their solvent capabilities, imidazolium-based ILs are also being explored as components in advanced materials, including electrolytes for batteries and supercapacitors [7], lubricants [8], and even in biomedical applications [9]. Within the epoxy resin chemistry, imidazolium-based ILs are increasingly being explored as both curing agents and additives [10,11,12]. Their ability to act as multifunctional components—acting as latent curing agents, enhancing the thermal and mechanical properties of the cured resins—positions them as innovative materials for advanced epoxy systems [13]. The integration of imidazolium-based ILs into epoxy resin systems has been heavily studied, with EMIM DCA and EMIM OAc being the most extensively studied curing agents [14].

Imidazolium-based ILs with long alkyl chains possess antimicrobial properties, even at low concentrations. The docusate anion (DOSS) also enhances the surface-active properties of ILs, making them useful in the formulation of emulsions, surfactants, and in processes that benefit from the reduction of surface tension [15]. Additionally, the presence of docusate can influence the viscosity and ionic conductivity of the ILs, which are critical factors in their performance as solvents, lubricants, or components in electrochemical devices [16]. The large, flexible structure of the docusate anion contributes to the formation of ILs with lower melting points, broadening their applicability across a range of temperatures.

Ionic liquids with docusate anions can offer additional benefits in materials chemistry, particularly in the development of epoxy resin-based composite materials, through their potential to impart antimicrobial properties. The amphiphilic nature of the docusate anion, combined with its surfactant-like behavior, can disrupt microbial cell membranes, leading to antimicrobial effects [17]. When incorporated into epoxy resin systems, these properties can be harnessed to create composite materials with inherent antimicrobial activity. The integration of docusate-based ILs into epoxy resins not only can enhance the physical and chemical properties of the material but also can add a functional layer of protection against microbial contamination. This dual functionality—enhancing both material performance and providing antimicrobial protection—positions docusate-based ILs as a multifunctional component in the development of advanced composite materials.

Research on the application of ILs in epoxy resin systems typically focuses on one of their functions within the system, such as (i) acting as a latent curing agent, (ii) flame retardant, or (iii) influencing the mechanical properties of the cured resin. Occasionally, the literature reports define the dominant—primary—function of an ionic liquid in a resin system and identify an additional function, such as impacting the mechanical properties of the composite. In such cases, the primary function is usually the one where significant change is observed, while the additional functions have a less pronounced impact. Even less frequently, research is conducted on mixtures of ionic liquids that, through the careful selection of individual components, could provide more than one significant function in a composite system.

The aim of this study is to investigate the effect of ionic liquid mixtures on the properties of epoxy resin. A dual design approach was adopted. In the first stage, the structures of individual ILs were designed with the goal of providing specific functionality: (i) activity as a curing initiator for the resin, ensured by the presence of a dicyanamide anion followed by an imidazole-based cation core, and (ii) an additive increasing the flexibility of the cured resin, planned to be achieved by introducing long alkyl chains into the cation structure or a bulky anion such as DOSS. This anion, due to its chemical properties, can not only influence the flexibility of the material but is also expected to exhibit a mild antimicrobial effect, although this is not the focus of this study and will be evaluated in future during the development of fiber-reinforced composite structures. The second design stage involved the evaluation of the molar ratios of the individual components of the mixtures, conducted through screening tests in a vacuum oven on samples with varying compositions and using the differential scanning calorimetry (DSC) technique. The goal of this stage was to achieve an appropriate balance between the various functions of the ILs mixture. During the conceptualization, a specific direction for the future application of this resin system was defined, and the crucial functionalities for achieving positive results in application-oriented research were identified. The defined application area objective includes the use of the tested resin system as a component in the manufacture of fiber-reinforced composite structures, specifically hybrid flax-carbon fibers, intended for structural elements of sports equipment such as skateboards, helmets, or surfboards. In this application area, the flexibility of the materials and the presence of natural reinforcing fibers, which ensure lightweight construction, are essential. The mild bactericidal effect may prevent the growth of microorganisms on the surface of sports equipment, for example, coming from the sweat of users.

In this study, the focus was primarily on the resin system, its optimization, and the characterization of the crosslinking process, initiated by the ionic liquid present in the form of a mixture of ILs, which also contains other ILs that are inactive as curing agents. In some of the other literature reports on IL mixtures used to modify epoxy resins, the IL responsible for curing is sometimes added to the resin earlier, while the IL mixture responsible for modifying the system parameters is added later [18]. In this study, the resin system is formed in a single step by mixing the ILs mixture with the epoxy resin in specific weight ratios. Future research plans include scaling up from laboratory research to development studies on fiber-reinforced composite structures, targeting structural elements of sports equipment, with appropriately tailored analysis and testing.

## 2. Results and Discussion

### 2.1. Synthesis and Properties of Ionic Liquids

**Figure 1:**
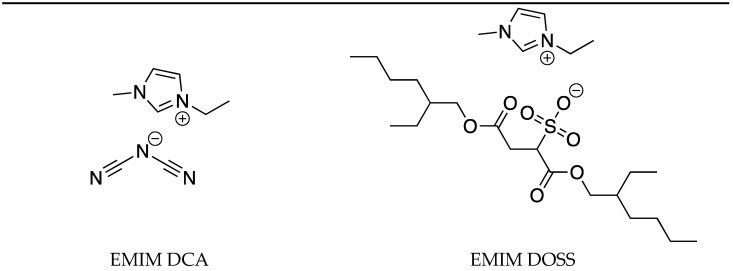

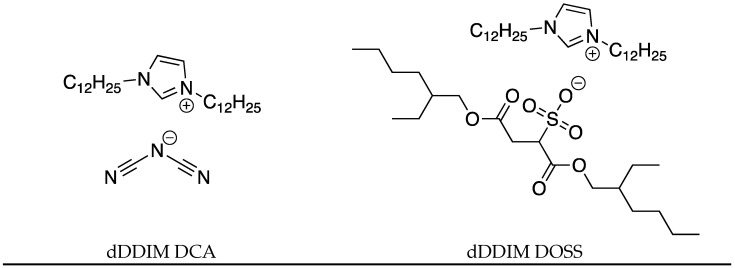
Chemical structures of ionic liquids used in this work.

In this work, four ionic liquids are used (Figure 1), and the synthesis of three of them is reported here for the first time. First, 1,3-didodecylimidazolium chloride was obtained. The adopted synthetic strategy involved a one-step dual substitution reaction. The imidazole was deprotonated with potassium bicarbonate, and two nitrogen atoms were substituted by two dodecyl carbon chains. The reaction was carried out for 7 days, and afterwards the workup procedure began. IL halides are known for their lack of miscibility in the non-polar solvents, such as hexane diethyl ether or ethyl acetate (which has some degree of polarity). However, because of the presence in the structure of two long carbon chains, this compound is slightly soluble in those solvents, especially when the after-reaction mixture is contaminated by the unreacted imidazole and/or dodecyl chloride. To achieve satisfactory reaction yield, key workup steps have been identified. After solvent evaporation, the mixture should be allowed to form a solid. This allows the ionic liquid to crystallize. Then, the resulting solid was washed with ethyl acetate, and after a few minutes the mixtures were separated, the white solid was washed with diethyl ether, and the solute (which gave a positive result with silver nitrate) was evaporated. The procedure was repeated three times. This method allowed to obtain pure salt with a 70% yield. Despite the workup protocol, this synthetic method is the most efficient in terms of substrate price, time, and purity.

Two anions were selected for this research: dicyanamide (DCA) and docusate. 1,3-Didodecylimidazolium dicyanamide [dDDIM DCA] was obtained with freshly prepared silver dicyanamide according to the well-established procedures [19] with very high yields and purities. On the other hand, DOSS salts were obtained through the metathesis reaction with docusate sodium salt. Chlorides were dissolved in acetone with sodium salt. The mixture was stirred for 24 h at room temperature. Afterwards, the resulting sodium chloride was filtered off, and the resulting solution was evaporated. The anion exchange was monitored by ion chromatography. Both DOSS ILs have purities above 99% and were obtained in almost equimolar ratios.

The thermal properties of the three ionic liquids (EMIM DOSS, dDDIM DCA, and dDDIM DOSS) reveal distinct behaviors as observed from the DSC analysis, and the results are presented in Table 1.

EMIM DOSS has the lowest T_g_ at −56.3 °C, indicating that it remains in a glassy, rigid state at much lower temperatures compared to the other ILs. In contrast, dDDIM DOSS, with a T_g_ of −43.9 °C, transitions to a rubbery state at a slightly higher temperature than EMIM DOSS, yet it still operates in a low temperature range. On the other hand, dDDIM DCA has a significantly higher T_g_ of 7.0 °C, which means it transitions to a rubbery or viscous state at a much higher temperature, making it potentially more rigid at lower temperatures than the other two ionic liquids. The cold crystallization temperature (T_cc_) is the temperature at which the ionic liquid crystallizes upon reheating after being cooled below its melting point without crystallization. This phenomenon is different from the crystallization temperature (T_c_), which is the temperature at which the ionic liquid crystallizes during the cooling process. For dDDIM DOSS, the T_cc_ is the lowest at −26.0 °C, indicating that it crystallizes at a lower temperature upon reheating, which might affect its thermal properties in cooling-reheating cycles. EMIM DOSS has a T_cc_ of −13.0 °C, higher than dDDIM DOSS but still lower than dDDIM DCA. dDDIM DCA has the highest T_cc_ at 9.0 °C, suggesting it has a higher tendency to crystallize upon reheating at a much higher temperature compared to the other two ionic liquids.

In terms of melting temperature (T_m_), dDDIM DCA shows the highest T_m_ at 41.2 °C, indicating that it requires more heat to transition from a solid to a liquid, suggesting higher thermal stability. On the other hand, dDDIM DOSS has a Tm of −1.4 °C. EMIM DOSS does not exhibit a distinct melting point in the provided data, which could suggest either a broad melting range or potential decomposition before a clear melting phase is observed.

Examining the crystallization temperature (T_c_) during the cooling process, dDDIM DOSS has the lowest T_c_ at −30.3 °C, which indicates that it crystallizes at a lower temperature upon cooling. EMIM DOSS, with a Tc of −25.8 °C, also tends to crystallize at relatively low temperatures. In contrast, dDDIM DCA has a Tc of 2.3 °C, which is much higher, reflecting a greater tendency to remain in a liquid state at lower temperatures.

### 2.2. Mixtures of Ionic Liquids

The ionic liquid mixtures were prepared with different molar ratios to evaluate curing properties of those mixtures (Table 2). 

The prepared systems consisted of two ILs, based on the same cation structure but different anions. As such, MIL1 and MIL3 are mixtures of equimolar amounts of, respectively, EMIM DCA and DOSS and dDDIM DCA and DOSS. MIL2 and MIL4 were prepared with higher molar concentrations of DCA salts, but with almost equal mass ratios. Lastly, MIL5 and MIL6 were prepared with 5 moles of DCA salts, and one mol of DOSS salts. MIL1 and MIL2 contain 1,3-Didodecylimidazolium dicyanamide (IL1) and 1,3-Didodecylimidazolium docusate (IL2), with the highest viscosities among the mixtures. This might be due to the long alkyl chains of IL1 and IL2, which tend to increase viscosity due to stronger intermolecular interactions and hinder molecular mobility. MIL3, MIL4, and MIL6 contain IL3 (1-Ethyl-3-methylimidazolium dicyanamide) and IL4 (1-Ethyl-3-methylimidazolium docusate). These mixtures have much lower viscosities compared to the IL1/IL2 systems. The shorter alkyl chains in IL3 and IL4 result in less molecular entanglement and weaker van der Waals interactions, which likely explains the lower viscosity values. Mixtures containing 1,3-Didodecylimidazolium-based ILs exhibit higher viscosities, and increasing the ratio of IL1, as seen in MIL5 (5:1 ratio), reduces viscosity slightly. Mixtures with 1-Ethyl-3-methylimidazolium-based ILs have much lower viscosities. MIL6, with the highest IL3 content, has the lowest viscosity (0.05 Pa·s), suggesting that dicyanamide anions with shorter alkyl chains lead to a more fluid system.

The thermal properties of the prepared mixtures are shown in Table 3.

MIL1 exhibits a complex thermal profile with multiple transitions, reflecting a combination of crystallization and melting behavior. The observed cold crystallization temperatures of −39.7 °C, −6.8 °C, and −39.6 °C, combined with melting points at −24.0 °C and −6.6 °C, suggest that MIL1 undergoes multiple phase changes at relatively low temperatures. This indicates a material that is likely to be semi-solid or soft at room temperature, with potential use in applications where flexibility in ambient conditions is required. The crystallization temperatures of 25.0 °C and −16.5 °C further support that MIL1 may retain some degree of order even as it transitions between different phases.

MIL2 also exhibits several phase transitions, although its thermal behavior differs from that of MIL1. The glass transition temperature of −18.9 °C indicates that MIL2 remains relatively flexible at lower temperatures. However, the presence of multiple cold crystallization temperatures (−10.7 °C and −22.3°C) and melting points (−4.3 °C and 7.9 °C) suggests that MIL2 may exhibit polymorphism, with different crystalline forms present. The crystallization temperature of 33.2 °C, above room temperature, suggests that MIL2 could solidify and retain a more structured form under certain conditions, making it suitable for applications that require a material that crystallizes at slightly elevated temperatures.

In contrast, MIL3 shows a significantly lower glass transition temperature of −67.7 °C with no recorded crystallization or melting transitions in the range tested. This suggests that MIL3 remains highly flexible and possibly amorphous over a wide temperature range, making it an ideal candidate for applications where low temperature performance is critical. The lack of crystallization or melting points indicates that MIL3 does not readily form a crystalline structure, contributing to its thermal stability and suitability for environments where a non-crystalline, stable phase is desired. MIL4 has no recorded thermal transitions, which could mean that it either does not undergo significant phase changes in the range tested or that the data was not captured. This lack of data makes it challenging to draw conclusions about its thermal behavior without further testing. MIL5 presents a more ordered thermal behavior, with a cold crystallization temperature of 2.2 °C and melting points at 38.9 °C and 19.2 °C. These transitions suggest that MIL5 could solidify at room temperature, leading to a more crystalline and less flexible material. The crystallization temperature of −6.5 °C supports this idea, indicating that MIL5 can form a solid structure even under slightly sub-ambient conditions. This mixture might be well suited for applications that require a defined crystalline phase at or near room temperature.

Finally, like MIL3, MIL6 has a very low glass transition temperature of −79.2 °C, near the end of the thermal cycle range. The absence of crystallization and melting transitions suggests that MIL6, like MIL3, is likely amorphous and retains its flexibility over a wide range of temperatures. This property makes MIL6 particularly useful in situations where thermal stability and flexibility are necessary, even at extremely low temperatures.

### 2.3. Epoxy Resin Polymerization with Ionic Liquids and Their Mixtures

First, the analysis of the thermal curing properties of individual ionic liquids was conducted with different concentrations, 6 and 12 phr (parts per hundred resin). The results can be found in Table 4.

**Figure 2:**
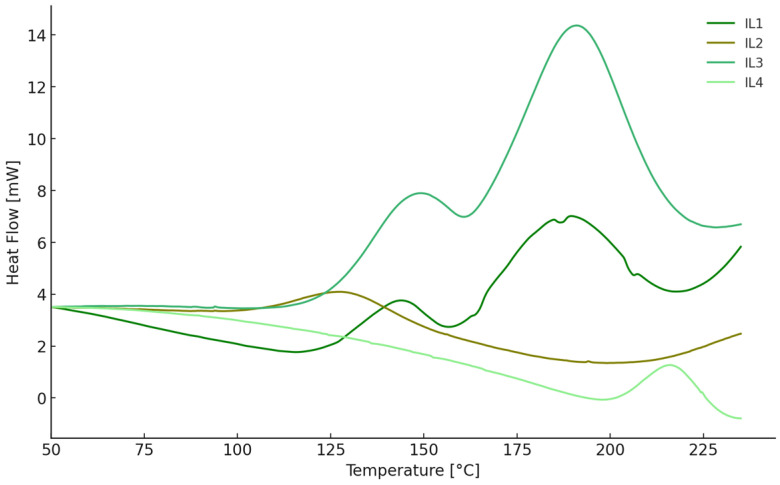
DSC thermograms for the polymerization of epoxy resin induced by ionic liquids (12 phr).

EMIM DCA exhibits distinct curing characteristics marked by the presence of two polymerization peaks at both 6 phr and 12 phr concentrations, indicating two polymerization pathways, initiated by the cation and anion of the IL. At 6 phr, it exhibits T_max_ values of 146 °C and 193 °C for its two polymerization peaks, with a total energy of the process given as an enthalpy, ΔH, of 98 J/g. Increasing the concentration to 12 phr does not alter the temperatures but leads to a substantial increase in ΔH to 184 J/g. In comparison, EMIM DOSS demonstrates a single-stage curing process without multiple peaks, probably the cationic pathway. Docusate salt displays much higher T_max_ values (214 °C and 216 °C for 6 phr and 12 phr, respectively), but these are coupled with very low ΔH values (8 J/g and 12 J/g). While the high T_max_ indicates that dDDIM DOSS requires higher temperatures to achieve a peak of maximum curing, the low enthalpy implies that the overall cure process is less energetic. Similarly, dDDIM DCA at 6 phr shows T_max_ values at 147 °C and 183 °C with a relatively low ΔH of 21 J/g. When the concentration is increased to 12 phr, T_max_ shifts slightly to 143 °C and 189 °C, and ΔH rises significantly to 55 J/g. In the case of the DCA anion in place of the DOSS anion, an increase in the total energy of the process is observed, indicating that the anion is also involved in the polymer network formation process. As the IL concentration increases, the energy increases but does not reach the value observed for EMIM DCA, which indicates a smoother crosslinking process, leading to a lower density of connections between resin adducts. Figure 2 presents thermograms for the curing process of epoxy resin with just ionic liquids with 12 phr.

Table 5 presents results obtained from DSC analysis for the curing of epoxy resin with the mixtures of ionic liquids.

**Figure 3:**
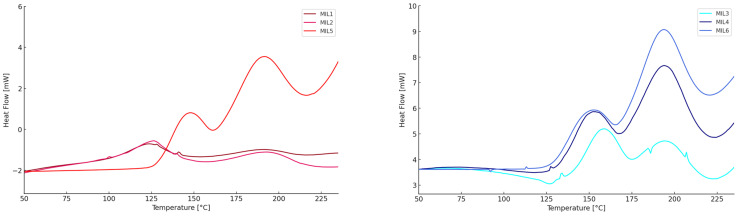
DSC thermograms for the polymerization of epoxy resin induced by 1,3-didodecylimidazolium-based ionic liquids 12 phr (**left**) and induced by 1-ethyl-3-methylimidazolium-based ionic liquids 12 phr (**right**).

Studies on the MIL1-M6 mixtures were carried out using the DSC technique, with two concentrations (6 and 12 phr) of the ILs mixture relative to the epoxy resin. Mixtures MIL1, MIL2, and MIL5 consist of ionic liquids dDDIM DCA and dDDIM DOSS in different molar ratios: 1:1, 2:1, and 5:1. In contrast, mixtures MIL3, MIL4, and MIL6 consist of ionic liquids EMIM DCA and EMIM DOSS in molar ratios of 1:1, 3:1 and 5:1. The systems were designed so that one of the components, assumed to be the IL with DCA anion, is responsible for the efficient curing process and the other one, which will modify the properties of the cured resin. The results present that the molar ratios of the individual ionic liquids in the mixture influence the parameters of the crosslinking processes. For mixtures MIL1, MIL2, and MIL5, both for 6 phr and 12 phr concentrations, we observe a significant increase in the process energy in terms of enthalpy as the concentration of the ionic liquid with the DCA anion in the mixture increases, obtaining values respectively of 4, 12, and 32 J/g for a concentration of 6 phr and 30, 36, and 96 J/g for a concentration of 12 phr, for mixtures in which the IL with the DCA anion was present in the amount of 1, 2, and 5 moles. In general, for mixtures MIL1, MIL2, and MIL5, the lower values of the molar ratios of the ionic liquid with the DCA anion to the ionic liquid with the DOSS anion in the mixture influence the lower temperature of the onset and peak of polymerization. On the other hand, in the MIL5 mixture in which the dDMIM-DCA ionic liquid is dominant, the temperature of the first peak of onset and peak of polymerization is 140 and 149 °C (6 phr) and 131 and 146 °C (12 phr), indicating that the dDMIM DOSS in the mixture is crucial to lower the process temperature and its molar ratio must be at a comparable level to that of IL with the DCA anion. Figure 3 presents DSC analysis for the polymerization process of epoxy resin for MIL1, MIL2, and MIL5 (shades of red on the left) and for MIL3, MIL4, and MIL6 (shades of blue on the right).

The mixtures MIL3, MIL4, and MIL6 consist of ionic liquids based on the 1-ethyl-3-methylimidazolium cation. In this case, unlike mixtures MIL1, MIL2, and MIL5, as the molar ratio of the liquid containing the DCA anion to the liquid containing the DOSS anion increases, a decrease in the temperature of the first peak of the polymerization onset and the temperature peak is observed, reaching the following values for the respective mixtures: 142, 141, and 136 °C (6 phr) and 141, 136, and 132 °C (12 phr). This suggests that, in the case of a simple cationic structure, an increase in the concentration of the IL with the DCA anion is desirable to lower the curing temperature of the epoxy resin. Similarly, also in the case of this group of IL mixtures, an increase in the total energy of the process is observed with the increase in the concentration of the ionic liquid with the DCA anion in the system, which ultimately indicates the correct identification of the component responsible for the effective curing of the epoxy resin. In addition to considering the molar ratios alone, it should be noted that the individual components of the mixtures differ significantly in terms of molecular weight, so these systems should also be considered from the perspective of the actual mass amount of individual ionic liquids in the systems and the weight fraction of DCA and DOSS anions in the resin systems. By adjusting the composition of the IL mixture, the crosslinking process can be precisely controlled according to the desired parameters.

Finally, in order to measure the hardness properties of epoxy resin cured with different systems, samples of four mixtures at the 12 phr concentration were prepared at three temperatures (120, 135, and 150 °C). The results can be found in Table 6.

MIL2, composed of a 1,3-Didodecylimidazolium dicyanamide (IL1) and 1,3-Didodecylimidazolium docusate (IL2) (2:1 molar ratio), shows a moderate increase in hardness as the curing temperature rises. The Shore A hardness increases from 50 at 120 °C to 65 at 150 °C, while the Shore D hardness increases from 30 to 40. This suggests that IL1, being more prevalent, promotes crosslinking in the epoxy network, but the overall hardness remains moderate, reflecting a balanced structure with some flexibility. MIL5 with 5:1 molar ratios of IL1 and IL2 demonstrates a significant increase in hardness with rising curing temperatures. The Shore A hardness rises from 70 at 120 °C to 86 at 150 °C, and the Shore D hardness increases from 36 to 48. MIL4 and MIL6, which are composed of EMIM DCA and EMIM DOSS in different ratios (3:1 and 5:1), maintain a constant Shore A hardness of 99 across all temperatures. However, MIL6 shows a more significant increase in Shore D hardness, from 62 at 120 °C to 85 at 150 °C. The consistently high Shore A hardness suggests a very hard surface due to the dominance of EMIM DCA. The elasticity and flexibility of MIL2 and MIL5 are influenced by the presence of long carbon chains in IL1 and IL2. MIL2, with its moderate Shore A hardness, retains more flexibility and elasticity, while MIL5, with higher Shore A values, becomes less flexible but still retains some elasticity due to the same structural features. The balance between the aliphatic chain length and the molar ratio of ionic liquids in these mixtures determines the tradeoff between rigidity and flexibility, allowing for the tailoring of mechanical properties to suit specific applications.

Table 7 presents the comparison between two papers published and this manuscript, which focuses on the applications of ionic liquid mixtures as curing agents in the epoxy resin polymerization. All three documents demonstrate that ionic liquid mixtures—particularly those based on dicyanamide anions—have a significant impact on the curing properties of epoxy resins. Across all studies, IL mixtures resulted in lower exothermic enthalpy and higher onset temperatures compared to traditional curing agents, promoting a more controlled and gradual curing process.

## 3. Materials and Methods

### 3.1. Materials

Reagents used within this paper were 1-ethyl-3-methylimidazolium chloride (Iolitec, 99%), 1-ethyl-3-methylimidazolium dicyanamide (Iolitec, Heilbronn, Germany, 99%), imidazole (Merck, Rahway, NJ, USA, 99%), dodecyl chloride (Merck, 99%), sodium dicyanamide (Ambeed, Arlington Heights, IL, USA, 99%), silver nitrate (Merck, 99%), docusate sodium salt (Ambeed, 99%), and potassium bicarbonate (Merck, 99%). Epoxy resin was LH 301. All solvents used were of HPLC grade.

### 3.2. Synthesis of Ionic Liquids

#### 3.2.1. 1,3-Didodecylimidazolium Chloride [dDDIM Cl]

Imidazole (10 g, 0.147 mol, 1.0 eq.) was dissolved in acetonitrile (50.0 mL), and the flask was placed under reflux and potassium bicarbonate (36.75 g, 0.368 mol, 2.5 eq.) was added in portions. Afterwards, the mixture was stirred for 30 min, and then dodecyl chloride (66.23 g, 0.323 mol, 2.2 eq., 76.12 mL) was added dropwise. The reaction was carried out for 7 days. First, the mixture was separated on the funnel, and the solution was evaporated. The obtained liquid was allowed to crystalize, and only afterwards was it was washed with ethyl acetate (3 × 100 mL). The white precipitate was formed and subsequently separated. Since the long alkyl chained ionic liquid is somewhat soluble in ethyl acetate, the remaining solution was evaporated, and the procedure was repeated 3 times. The white solid was also washed with diethyl ether. White solid was dried under vacuum. Yield: 70.0%, water content: 0.55 wt %, IC: t_R_ = 5.83 min (anion), purity: 99.5%. ^1^H NMR (400 MHz, CDCl_3_): δ = 10.43 (s, 1H), 7.27 (d, *J* = 1.5 Hz, 2H), 4.35 (t, *J* = 7.5 Hz, 4H), 2.63 (d, *J* = 10.4 Hz, 2H), 1.92 (p, *J* = 7.2 Hz, 4H), 1.34 (dd, *J* = 7.2 Hz, *J* = 3.9 Hz, 8H), 1.24 (s, 28H), 0.86 (t, *J* = 7.1 Hz, 6H).

#### 3.2.2. 1,3-Didodecylimidazolium Docusate [dDDIM DOSS]

1-3-Didodecylimidazolium chloride (20 g, 0.045 mol, 1.0 eq.) and docusate sodium salt (19.15 g, 0.045 mol, 1.0 eq.) were dissolved in acetone (100 mL) and stirred at room temperature for 24 h. Afterwards, the mixture was filtered off, and the remaining solution was evaporated. The obtained yellow liquid was dried under vacuum. Yield: 98.0%, water content: 0.24 wt %, IC: t_R_ = 22.91 min (anion), purity: 99.3%. ^1^H NMR (400 MHz, CDCl_3_): δ = 12.82 (s, 1H), 12.19 (d, *J* = 1.0 Hz, 2H), 9.34 (t, *J* = 7.0 Hz, 3H), 9.06 (ddd, *J* = 10.9 Hz, *J* = 5.3 Hz, *J* = 2.2 Hz, 4H), 8.83 (dd, *J* = 11.5 Hz, *J* = 3.6 Hz, 1H), 8.10 (dd, *J* = 17.2 Hz, *J* = 11.5 Hz, 1H), 7.98 (dd, *J* = 17.2 Hz, *J* = 3.7 Hz, 1H), 7.68 (p, *J* = 1.8 Hz, 3H), 6.96 (p, *J* = 7.1 Hz, 3H), 6.66 (tdd, *J* = 9.7 Hz, *J* = 6.8 Hz, *J* = 4.5 Hz, 2H), 6.42 (m, 48H), 6.02 (m, 18H).

#### 3.2.3. 1-Ethyl-3-Methylimidazolium Docusate [EMIM DOSS]

1-Ethyl-3-methylimidazolium chloride (20 g, 0.136 mol, 1.0 eq.) and docusate sodium salt (57.5 g, 0.136 mol, 1.0 eq.) were dissolved in acetone (100 mL) and stirred at room temperature for 24 h. Afterwards, the mixture was filtered off, and the remaining solution was evaporated. The obtained transparent liquid was dried under vacuum. Yield: 98.5%, water content: 0.21 wt %, IC: t_R_ = 21.53 min (anion), purity: 99.5%. ^1^H NMR (400 MHz, CDCl_3_): δ = 13.07 (s, 1H), 11.72 (t, *J* = 1.8 = Hz, 1H), 11.63 (t, *J* = 1.8 Hz, 1H), 8.13 (q, *J* = 7.3 Hz, 2H), 7.81 (m, 4H), 7.78 (s, 3H), 7.59 (dd, *J* = 11.5 Hz, *J* = 3.7 Hz, 1H), 6.85 (dd, *J* = 17.2 Hz, *J* = 11.5 Hz, 1H), 6.73 (dd, *J* = 17.2 Hz, *J* = 3.7 Hz, 1H), 7.43 (p, *J* = 1.9 Hz, 1H), 5.42 (m, 2H), 5.34 (t, *J* = 7.4 Hz, 3H), 5.24 (m, 4H), 5.17 (m, 12H), 4.76 (m, 12H).

#### 3.2.4. 1,3-Didodecylimidazolium Dicyanamide [dDDIM DCA]

1-3-Didodecylimidazlium dicyanamide was obtained according to the procedure previously reported by us [19]. Briefly, freshly prepared silver dicyanamide was used with the water solution of the corresponding chloride. The resulting precipitate was filtered off and the solution was evaporated. The obtained white solid was dried under vacuum. Yield: 99.0%, water content: 0.32 wt %, IC: t_R_ = 14.22 min (anion), purity: 99.4%. ^1^H NMR (400 MHz, CDCl_3_): δ = 13.13 (s, 1H), 11.73 (d, *J* = 1.6 Hz, 2H), 8.09 (t, *J* = 7.0 Hz, 4H), 5.72 (p, *J* = 7.1 Hz, 4H), 5.17 (s, 36H), and 4.78 (t, *J* = 6.7 Hz, 6H).

### 3.3. Mixture Preparation

Four ionic liquids were used to prepare cross systems for the epoxy resin. Molar ratios of ionic liquids used to prepare mixtures are presented in Table 2. Discussed systems were prepared by stirring at elevated temperature (60 °C) for 2 h. Afterwords, the mixtures were dried under vacuum.

### 3.4. Ion Chromatography (IC)

IC measurements were conducted in accordance with the procedures published in our previous works [21].

### 3.5. Water Content Analysis

Water content was determined using Karl Fischer-type titration apparatus according to method reported in previous work [22].

### 3.6. Differential Scaning Calorimetry (DSC)

DSC analysis for ionic liquids and their mixtures as well as the curing properties of ionic liquids and their mixtures were conducted according to the procedure published in our previous works.

### 3.7. NMR Analysis

NMR spectra were recorded using a 400 MHz (^1^H) Bruker Ascend spectrometer (Bruker Corporation, Billerica, MA, USA) in a commercially available CDCl_3_ solvent. The value of standard measurement uncertainty did not exceed ± 0.02 ppm.

### 3.8. Curing Proces

All experiments related to the curing process were carried out in accordance with the procedure detailed in our previous work [23].

### 3.9. Hardness Measurements

All hardness measurements were conducted following the procedure outlined in our previous work [23].

### 3.10. Viscosity Measurements

Viscosity measurements were conducted using rotary rheometer according to the procedure detailed in our previous work [22].

## 4. Conclusions

The research presented in this study successfully synthesized and characterized three ionic liquids. These ILs, with their unique thermal properties and varied anion-cation combinations, were further explored in mixtures to assess their potential in epoxy resin polymerization. The thermal analysis revealed significant differences in glass transition, crystallization, and melting temperatures among the synthesized ionic liquids, highlighting their diverse thermal stability and flexibility. For instance, dDDIM DCA exhibited a higher thermal stability with a T_g_ of 7.0 °C, whereas the other ILs demonstrated lower T_g_ values, indicative of greater flexibility at lower temperatures. Mixtures MIL3 and MIL6, which contain higher concentrations of DCA ionic liquid, exhibited very low T_g_ values (−67.7 °C and −79.2 °C, respectively), indicating a highly flexible and possibly amorphous state over a wide temperature range. The absence of distinct crystallization or melting transitions suggests that these mixtures maintain their flexibility and thermal stability, making them ideal for low-temperature applications where a non-crystalline phase is advantageous. MIL1 and MIL2 showed multiple thermal transitions, indicating complex phase behaviors. The study of ILs mixtures showed that the molar ratio of the constituent ionic liquids significantly impacts the curing behavior of epoxy resins. Mixtures with higher concentrations of the DCA anion, particularly those based on EMIM, exhibited increased enthalpy values and lower onset polymerization temperatures, indicating more efficient curing processes. Furthermore, the mechanical testing demonstrated that the hardness of the cured epoxy resin can be modulated by adjusting the curing temperature and the composition of the ionic liquid mixture. Mixtures containing EMIM DCA and EMIM DOSS displayed high Shore A hardness across all tested temperatures, indicating a potential for applications requiring hard, durable surfaces. In contrast, mixtures with a higher proportion of dDDIM DCA showed a balance between rigidity and flexibility, making them suitable for applications where both mechanical strength and elasticity are needed. In conclusion, the findings of this study underscore the versatility of ionic liquids in tailoring the thermal and mechanical properties of epoxy resins. By varying the anion-cation combinations and their ratios, it is possible to fine-tune the curing process and the resulting material properties, making these ionic liquids promising candidates for advanced material design in a wide range of industrial applications.

## Figures and Tables

**Table 1:** Thermal properties of ionic liquids.

IL	T_g_ (°C)	T_cc_ (°C)	T_m_ (°C)	T_c_ (°C)
EMIM DOSS	−56.3	−13.0		−25.8
dDDIM DCA	7.0	9.0	41.2	2.3
dDDIM DOSS	−43.9	−26.0	−1.4	−30.3

T_g_—glass transition temperature; T_cc_—cold crystallization temperature; T_m_—melting temperature; T_c_—crystallization temperature.

**Table 2:** Molar ratios of ionic liquids used to prepare curing systems for epoxy resin and their viscosities.

Mixture	Viscosity (Pa⋅s)	IL1	IL2	IL3	IL4
MIL1	0.46	1	1		
MIL2	0.21	2	1		
MIL3	0.13			1	1
MIL4	0.07			3	1
MIL5	0.10	5	1		
MIL6	0.05			5	1

IL1—1,3-Didodecylimidazolium dicyanamide; IL2—1,3-Didodecylimidazolium docusate; IL3—1-Ethyl-3-methylimidazolium dicyanamide; IL4—1-Ethyl-3-methylimidazolium docusate.

**Table 3:** Thermal properties of ionic liquid mixtures.

Mixture	T_g_ (°C)	T_cc_ (°C)	T_m_ (°C)	T_c_ (°C)
MIL1		−39.7	−24.0 25.0	−16.5 −6.8 −6.6 −39.6
MIL2	−18.9	−10.7 −4.3	33.2	7.9 −22.3
MIL3	−67.7			
MIL4				
MIL5		2.2	38.9	19.2 −6.5
MIL6	−79.2 *			

T_g_—glass transition temperature; T_cc_—cold crystallization temperature; T_m_—melting temperature; T_c_—crystallization temperature; *—temperature close to the cycle end temperature.

**Table 4:** Thermal curing properties of ionic liquids with epoxy resin.

Ionic Liquid	T_onset_ (°C)	T_max_ (°C)	T_endset_ (°C)	ΔH (J/g)
EMIM DCA 6 phr *	129/169	146/192	160/214	98
EMIM DCA 12 phr *	131/167	146/191	166/214	184
EMIM DOSS 6 phr	202	214	225	8
EMIM DOSS 12 phr	203	216	228	12
dDDIM DCA 6 phr *	136/164	147/183	155/203	21
dDDIM DCA 12 phr *	126/165	143/189	164/208	55
dDDIM DOSS 6 phr	96	117	133	11
dDDIM DOSS 12 phr	107	129	148	18

T_onset_—start polymerization temperature; T_max_—peak polymerization temperature; T_endset_—end polymerization temperature; ΔH—enthalpy; *—two polymerization peaks observed.

**Table 5:** Thermal curing properties of ionic liquid mixtures with epoxy resin.

Mixture	T_onset_ (°C)	T_max_ (°C)	T_endset_ (°C)	ΔH (J/g)
MIL1 6 phr	129/150	130/150	131/151	4
MIL1 12 phr	100/164	123/190	144/211	30
MIL2 6 phr	105/171	129/185	145/205	12
MIL2 12 phr	106/165	127/194	144/216	36
MIL3 6 phr *	142	160	172	12
MIL3 12 phr	141/187	158/196	173/209	33
MIL4 6 phr	141/169	153/189	163/209	31
MIL4 12 phr	136/173	152/194	165/213	81
MIL5 6 phr	140/165	149/186	159/208	32
MIL5 12 phr	131/167	146/190	160/212	96
MIL6 6 phr	136/169	150/191	162/212	60
MIL6 12 phr	132/170	149/193	165/214	142

T_onset_—start polymerization temperature; T_max_—peak polymerization temperature; T_endset_—end polymerization temperature; ΔH—total enthalpy; *—only one polymerization peak observed.

**Table 6:** Hardness analysis of obtained epoxy resin materials.

Curing Temperature (°C)	MIL2		MIL4		MIL5		MIL6	
Shore Type	A	D	A	D	A	D	A	D
120	50	30	99	84	70	36	99	62
135	58	37	99	84	76	43	99	70
150	65	40	99	85	86	48	99	85

**Table 7:** Comparison of the results between the three papers, that focused on the mixtures of ionic liquids and their curing properties.

Property	Manuscript	Publication 1 [18]	Publication 2 [20]
Ionic Liquids Studied	Imidazolium-based ILs with docusate and dicyanamide anions	ILs with phosphorus atom in the structure (cation and/or anion), IL with silicon atom in the structure and EMIM DCA	DCA-based ILs with long carbon alkyl chains in the cation structure
Amount of Ionic Liquids Used	6–12 wt%	12 wt%	4 wt%
Onset Polymerization Temperature	Two polymerization peaks 100−170 °C	110–133 °C	Two polymerization peaks 160 °C–185 °C
Peak Polymerization Temperature	Two polymerization peaks 123–196 °C	121–169 °C	Two polymerization peaks 170–200 °C
Enthalpy (ΔH)	Moderate (lower than traditional curing agents but higher than single IL systems)	High with values ranging from 446 to 508 J/g	significantly lower than single IL systems
Polymerization Behavior	Slower, controlled polymerization with moderate exothermic release	Lower exothermic release, gradual polymerization	Most controlled polymerization process with lowest exothermic reaction
Comparison to Single IL Systems	Lower enthalpy and slower curing than single ILs	Reduced enthalpy compared to single IL systems and conventional curing agents	Lowest enthalpy and onset temperatures compared to single ILs
Unique Findings	Focus on mixtures with docusate and dicyanamide anions, dual polymerization peaks observed, moderate enthalpy	Multifunctional ILs affecting various properties; lower enthalpy; curing and flexibility improvement	Dual polymerization peaks observed, indicating parallel reactions; extremely low enthalpy; controlled curing

## Data Availability

The raw data supporting the conclusions of this article will be made available by the authors on request.
